# Synthesis, Structure, Properties And Biological Behaviour Of The Complex [Ru^IV^ (H_2_L) Cl_2_].2H_2_O (H_4_L= 1,2-Cyclohexanediamminetetraacetic Acid)

**DOI:** 10.1155/S1565363304000160

**Published:** 2004

**Authors:** Rosario A. Vilaplana, A. Castiñeiras, Francisco González-Vílchez

**Affiliations:** 1 Departamento de Química Inorgánica Sección de Química Bioinorgánica Facultad de Química, Aptdo. Correos 553 Sevilla E-41071 Spain; 2 Departamento de Química Inorgánica Facultad de Farmacia Universidad de Santiago de Compostela Santiago de Compostela E-15782 Spain

## Abstract

The highly water-soluble ruthenium complex [Ru(H_2_L)Cl_2_]_2_H_2_o, in which H_4_L is the sequestering ligand trans-l, 2-cyclohexanediamminetetraacetic acid (cdta) has been synthesized, structurally characterized and its properties studied. The X-ray crystallographic study shows that the chelating coordinated ligand is tetradentate while the ruthenium environment is octahedral and slightly distorted, with two chloride anions coordinated in cis positions. Potentiometric, conductimetric and infrared studies confirm the presence of two free carboxylic groups, while electronic and voltammetric studies show that the central ion is Ru(IV). The testing of the cytotoxic activity of this complex against three different human cancer cell lines indicates that [Ru(H_2_L)Cl_2_].2H_2_O shows a remarkable and selective antiproliferative effect against the human uterine neck carcinoma HeLa and the malign adenocarcinoma ADLD, showing only a discrete turnout cell inhibition activity against colon adenocarcinoma HT-29. The important antiprotiferative behaviour of complex 1 against the human adenocarcinoma ADLD, indicates that [Ru(H_2_L)Cl_2_].2H_2_O might be considered as potential antineoplastic compound.


### Synthesis, Structure, Properties And Biological Behaviour Of The Complex [Ru $^{IV}$ ($H_2L$) $Cl_2$] · $2H_2O$ ($H_4L$ = 1,2-Cyclohexanediamminetetraacetic Acid)

Rosario A. Vilaplana,[a] A. Castiñeiras[b] and Francisco González-Vílchez *,[a]

[a]*Departamento de Química Inorgánica, Sección de Química Bioinorgánica, Facultad de Química, Aptdo. Correos 553, E-41071 Sevilla, Spain.*

[b]*Departamento de Química Inorgánica, Facultad de Farmacia, Universidad de Santiago de Compostela, E-15782 Santiago de Compostela, Spain*

**GRAPHICAL ABSTRACT**

The octahedral complex [Ru($H_2$cdta)$Cl_2$]·$2H_2O$ contains Ru(IV) surrounded by two nitrogen atoms, N(1) and N(2), and two chloride ions *cis* to each other, all in the equatorial plane. Two oxygen atoms, O(11) and O(21) are located in *trans* axial octahedral positions. The complex shows remarkable *in vitro* and *in vivo* activity against different carcinomas.

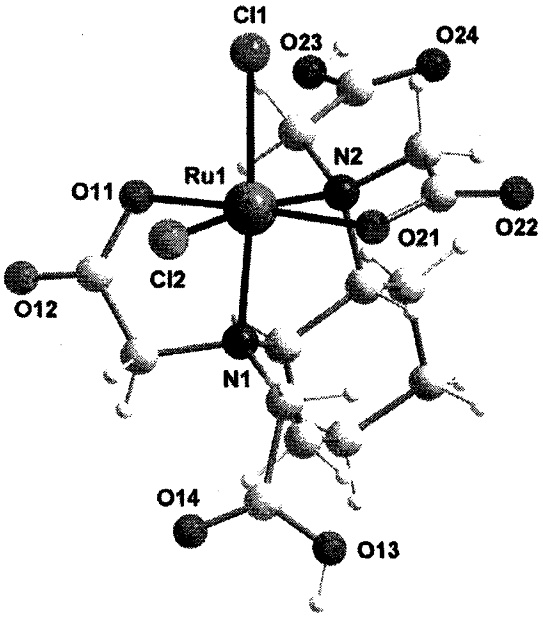

*to whom correspondence should be addressed; phone: +34 95 4557159; fax: +34 95 4557081; e-mail: fgonzalezv@us.es

### ABSTRACT

The highly water-soluble ruthenium complex $[Ru(H_2L)Cl_2]\cdot 2H_2O$, in which $H_4L$ is the sequestering ligand trans-1,2-cyclohexanediamminetetraacetic acid (cdta) has been synthesized, structurally characterized and its properties studied. The X-ray crystallographic study shows that the chelating coordinated ligand is tetradentate while the ruthenium environment is octahedral and slightly distorted, with two chloride anions coordinated in *cis* positions. Potentiometric, conductimetric and infrared studies confirm the presence of two free carboxylic groups, while electronic and voltammetric studies show that the central ion is Ru(IV). The testing of the cytotoxic activity of this complex against three different human cancer cell lines indicates that $[Ru(H_2L)Cl_2]\cdot 2H_2O$ shows a remarkable and selective antiproliferative effect against the human uterine neck carcinoma HeLa and the malign adenocarcinoma ADLD, showing only a discrete tumour cell inhibition activity against colon adenocarcinoma HT-29. The important antiproliferative behaviour of complex **1** against the human adenocarcinoma ADLD, indicates that $[Ru(H_2L)Cl_2]\cdot 2H_2O$ might be considered as potential antineoplastic compound.

### INTRODUCTION

Metal-based antitumour drugs constitute in our days a broad research area of increasing interest /1-4/. Concretely, cisplatin, cis-$[Pt(NH_3)_2Cl_2]$, carboplatin [cis-diammine-1,1-cyclobutanedicarboxylato-platinum(II)] and oxaliplatin [trans-(R,R)-1,2-diamminecyclohexaneoxalatoplatinum (II)], are currently being used clinically /5-10/. However, the problem of both acquired and inherent resistance of tumour cells presents a main limitation to the more widespread clinical use of platinum complexes /3,7,9/. In the hope of overcoming these limitations, other platinum-based antitumour drugs have been synthesized and tested for antitumour activity /11-15/. Furthermore, new anticancer drugs containing transition metal ions other than platinum /16/ have been also assayed. Possible advantages may involve different coordination geometries, several metal-ion oxidation states and biological targets other than DNA.

In the design of these new drugs, ruthenium complexes have raised great interest /17-24/. As an example, the antitumour activity of the highly water soluble complex $H[Ru(H_2L)Cl_2]\cdot 4H_2O$ ($H_4L$: 1,2-propylenediammine-N,N,N',N'-tetraacetic acid, pdta) has been evaluated *in vivo* and *in vitro* /23,25/. This complex rapidly binds to serum proteins producing stable adducts in which the core Ru(III)(pdta) is probably bound to histidines on the protein surface /26-28/. On the other hand, the complex damages nuclear DNA, inhibits DNA recognition and stimulates NADPH oxidase and a respiratory burst in phagocytic neutrophils and elicits phosphorylation of tyrosine residues /25,29/.

Another interesting ligand is the potential hexadentate sequestering agent *trans*-1,2-cyclohexanediamminetetraacetic acid (cdta), in which the ethylenediammine group of edta has been replaced by the heterocyclic moiety *trans*-1,2-cyclohexanediammine. The ligand cdta is widely used nowadays in varied applications, *i.e.*, as detoxifier of heavy metals that contaminate patients /30/, for use in endodontics /31/, for extraction of pectin polymers /32/ and for separation and quantitation by several techniques of inorganic ions and anionic metal complexes /33/. Although the metal complexes formed by cdta are known from time ago /34/, recent literature has shown new synthesis procedures and important properties of new isolated complexes. Concretely, those formed by cdta with Cu(II) and Ni(II) have been studied by X-ray photoelectron spectroscopy and X-ray crystal diffraction studies /35,36/ and interesting results on the structure, electrochemistry, kinetics, $pK_a$ values and influence of chelate effects on the water-exchange mechanisms of complexes cdta/Fe(III)/Fe(II)/ have been reported /37,38/. Research on the formation constants and dissociation kinetic of cdta/lanthanide(III)/ complexes /39,40/ as well as solution studies and determination of the crystal structures of cdta complexes formed with the trivalent ions of Al, Ga, In and Sc /41/ have been also published.

The research developed so far on platinum-group metal complexes formed with cdta concerns first the the X-ray crystallographic study of the diprotonated ligand and its complexes with $[PdCl_4]^{2-}$ and $[PtCl_4]^{2-}$ salts /42a/. Additionally, the reaction kinetic processes involved in the formation of Pd(II) and Pt(II) complexes of different composition with amminepolycarboxylate ligands (*i.e.*, $\{(H_6L)[Pd/PtCl_4]\}$ or $[Pd(H_2L)]$) were also studied /42b/. Dinitrogen complexes formed between Ru(II) and cdta were isolated and characterized /42c/. On the other hand, promising results were obtained in the first study developed on the antitumour activity of new Pd(II)(cdta) complexes /42d/. Different Ru(III) complexes such as $[Ru(H_4L)Cl]^{3-n}$ and $[Ru(HL)Cl]^{3-(n-1)}$, were synthesized and characterized by analytical and electrochemical techniques /43/. Finally, reduction of molecular nitrogen to ammonia in aqueous solution under ambient conditions occurs in the presence of an illuminated $RuO_2$/Pt/CdS system; this reaction is catalysed by several Ru(II) complexes as, for example, $[Ru(cdta)N_2]^{2-}$ /44/.

As a result of the attractive research in progress on biological properties of complexes formed by Ru(III) with amminepolycarboxylic ligands, as well as the absence of structural and antitumour activity of Ru(cdta) complexes, we present in this paper the synthesis, characterization by chemical and spectroscopic techniques, x-ray crystal structure and biological behaviour of the water-soluble complex $[Ru(H_2L)Cl_2]\cdot 2H_2O$ (**1**), in which the ligand cdta ($H_4L$) is acting as tetradentate molecule /45/. Our results demonstrate that the complex contains Ru(IV) and shows activity against several *in vitro* and *in vivo* tumours. This research open a new way for the medical and pharmaceutical development of potential antineoplastic complexes formed by the chelating agent cdta with platinum metals.

### EXPERIMENTAL

### X-ray crystallography

A prismatic yellow-amber crystal of compound **1** was mounted (glass fiber) on an Enraf Nonius CAD4 automatic diffractometer /46/ and 2388 unique reflections were measured. Cell constants and orientation matrix for data collection were obtained by least-squares refinement of the $2\theta$ values of 25 reflections. Intensities of 3123 reflections within the range $2° < 2\theta < 50°$ were measured and collected at 293 K using monochromatic $MoK_\alpha$ radiation ($\lambda$ = 0.71073 Å) and the $\omega/2\theta$ scan technique. Intensities were corrected for Lorentz and polarization effects /47/ and 2388 $\{(I>2\sigma(I))\}$ were considered as observed. A semiempirical absorption correction (φ-scans) was made /48/.

The structure was solved by the Patterson method /49/ and subsequent difference Fourier maps, and refined on $F^2$ by a full matrix least-squares procedure using anisotropic displacement parameters /50/. All hydrogen atoms were located in difference map and included as fixed contributions riding on attached atoms with isotropic thermal parameters 1.2 times those of their carrier atoms. The H atoms of two water molecules, O(1) and O(2), were not located. Therefore, the contribution of the density of a disordered water molecule was subtracted from the measured structure factors with use of the SQUEEZE option /51/. Subsequent refinement then converged with *R* factors and parameter errors significantly better than for all attempts to model the solvent disorder. The Flack *x* parameter (absolute structure parameter) was calculated to be 0.05(6) for the present structure and 0.95(6) for the inverted structure, thus providing strong evidence that the absolute structure has been assigned correctly /52/. Criteria of a satisfactory complete analysis were the ratios of rms shift to standard deviation less than 0.001 and no significant features in final difference maps. Atomic scattering factors, from "International Tables for Crystallography" /53/. Molecular graphics, from PLATON /51/ and SCHAKAL /54/.

### Chemicals

Hydrated ruthenium (III) chloride (Sigma) was dissolved in ethanol and refluxed for 30 min. After concentration to dryness, the compound was stored under $CaCl_2$ ($RuCl_3$). The ligand cdta was used as purchased (Sigma). All other chemicals and solvents were analytical grade reagent products.

### Analytical, potentiometric and conductimetric studies

Elemental microanalyses were performed at the Microanalytical Laboratory of the Barcelona University. Metal content was determined by atomic absorption spectroscopy using a Perkin Elmer 2380 model, at 10 mA and $\lambda$ = 349.9 nm. Hydration water molecules were determined by thermal analysis. Potentiometric and conductimetric studies were carried out with a Crison MicroTT 2022 titrimeter, provided with autoburette Microbur 2030. Aqueous solutions of the complex (50-100 mg/100 ml) were titrated against a 30 mM NaOH solution. Electrical conductimetry of the same solution was performed on a Crison 525 conductimeter.

### Electronic and infrared spectroscopy

The electronic spectra of solutions were recorded on a Jasco V550 spectrometer interfaced with a PC. IR spectra were recorded on an FT-IR Jasco 300E instrument in the 200-4000 $cm^{-1}$ range, using either Nujol mulls supported between polyethylene plates or KBr pellets.

### Voltammmetric studies

Cyclic voltammetry measurements were carried out using a Princeton Applied Research analyser. A

glassy carbon electrode was used as working electrode on aqueous solutions ($NaClO_4$ 0.15 M) of complex **1** at concentrations ranging between 1.0 and 6.0 mM. Potentials were measured against a saturated (NaCl) calomel electrode (SCE) as reference electrode. Voltammograms (CV) were obtained at potential values between +1.5 and -1.5 V. Scan rate was equal to 50 mV/s.

### Biological *in vitro* and *in vivo* assays

Complex **1** was dissolved in a mixed 1:1 DMSO:$H_2O$ solution and used as concentrated stock solution from which diluted solutions (1/10 dilution factor) were prepared and added to growing culture medium of each one of the cellular types included in the study. The *in vitro* assays were done against three different human cancer cell cultures: malignant melanoma (ADLD), uterine neck carcinoma (HeLa) and colon adenocarcinoma (HT-29). For determination of plating efficiency and colorimetric reading, we prepared plates of 24 and 96 wells, respectively, that were then inoculated with 5,000 to 30,000 cells/well. After 24 h from the inoculation, different concentrations of the testing product were added to the cancer cells. After 48 h of the named addition, the cells were washed and then fixed. Finally, the coloring and reading of the plates were carried out. In all cases we determined the number of cells at the beginning ($T\alpha$), just before the addition of the testing products ($T_0$) and 48 h later (T controls and T tests), according to Scheme 1.

The antitumour activity of **1** was tested *in vivo* against Ehrlich ascitic tumour (EAT), the intraperitoneally-implanted P388 lymphocytic leukemia and the subrenal capsule (transplanted human mammary carcinoma) MX-1 xenograft. The experiments were performed according to the NIH protocols at ONI Centre, Madrid, Spain. $CD_2F_1$ female mice with weights within a 3 g value range and a minimum weight of 18 g were used for all experiments except for MX-1 carcinoma, where athymic swiss mice were used (4 g value range and weight of 17 g). The test groups was formed by 6 animals and control group by 12.

### Synthesis of the complex $[Ru^{IV}(H_2L)Cl_2]\cdot 2H_2O$ [1]

Solid cdta (228 mg, 1.0 mmol) was added to a clear solution of $RuCl_3$ (0.230 mg, 1.1 mmol) dissolved in a 50 ml of HCl 0.1 M, while stirring. The deep red mixture was introduced into a sealed pressure reactor and heated for 16 h at 120°C in electric oven. After cooling, the pink-red obtained solution was slowly concentrated to about 5 ml by evaporation at room temperature. Yellow-amber prismatic crystals suitable for X-ray diffraction studies were recovered and the solid analysed. Two hydration water molecules per mol of compound were found by thermal analysis. *Anal.* (%), Calc. for $C_{14}H_{20}O_8N_2Cl_2Ru\cdot 2H_2O$: C, 30.4; H, 4.5; N, 5.1; Cl, 12.8; Ru, 18.3. Found: C, 30.6; H, 4.1; N, 5.3; Cl, 12.2; Ru, 17.9.

### RESULTS AND DISCUSSION

### X-ray structure

Table 1 presents a summary of the relevant crystal data and refinement results for complex **1**. The

**Table 1**

Crystal data and structure refinement for $[Ru(H_2cdta)Cl_2]\cdot 2H_2O$

| | |
|---|---|
| Identification code | rudcta |
| Empirical formula | C14 H24 Cl2 N2 O10 Ru |
| Formula weight | 552.32 |
| Temperature | 293(2) K |
| Wavelength | 0.71073 A |
| Crystal system, space group | Hexagonal, P6(5) (No. 170) |
| Unit cell dimensions | a = 13.567(2) A alpha = 90 deg.<br>b = 13.567(2) A beta = 90 deg.<br>c = 22.286(6) A gamma = 120 deg. |
| Volume | 3552.5(12) A^3 |
| Z, Calculated density | 6, 1.549 Mg/m^3 |
| Absorption coefficient | 0.935 mm^-1 |
| F(000) | 1680 |
| Crystal size | 0.40 x 0.34 x 0.20 mm |
| Theta range for data collection | 1.73 to 27.44 deg. |
| Limiting indices | -14<=h<=0, 0<=k<=17, 0<=l<=28 |
| Reflections collected / unique | 3123 / 2779 [R(int) = 0.0103] |
| Completeness to theta = 27.44 | 100.0 % |
| Absorption correction | Psi-scans |
| Max. and min. transmission | 0.8350 and 0.7061 |
| Refinement method | Full-matrix least-squares on F^2 |
| Data / restraints / parameters | 2779 / 1 / 271 |
| Goodness-of-fit on F^2 | 0.943 |
| Final R indices [I>2sigma(I)] | R1 = 0.0498, wR2 = 0.0518 |
| R indices (all data) | R1 = 0.0498, wR2 = 0.0518 |
| Absolute structure parameter | 0.05(6) |
| Largest diff. peak and hole | 0.515 and -0.380 e.A^-3 |

molecular structure obtained by X-ray diffraction analysis is shown in Fig. 1 along with the numbering of atoms in the molecule. The tetradentate chelating ligand (cdta) contains two free carboxylic groups and is bonded to the central ion by two nitrogen atoms, N(1) and N(2), and two chloride anions *cis* to each other, sharing the equatorial plane with the nitrogen atoms. The remaining coordination positions are occupied by two oxygen atoms, O(11) and O(21), from coordinated carboxylate groups, situated in *trans* axial octahedral positions that complete in this way the octahedral coordination sphere around ruthenium atom.

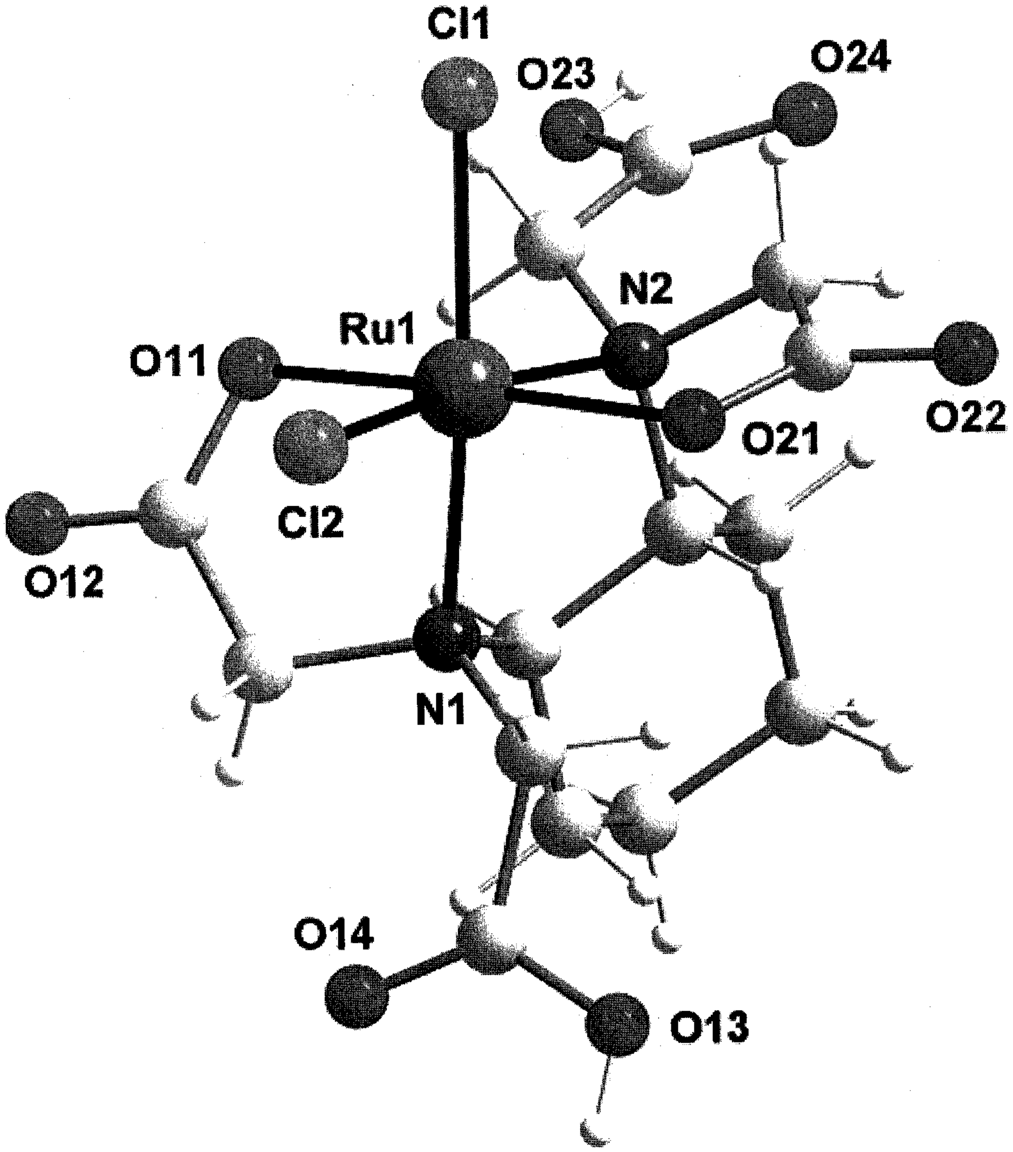

**Fig. 1:** View of the molecular structure of complex **1** showing the octahedral environment of the ruthenium central ion.

Table 2 presents selected bonds lengths and angles for complex **1**. As expected, the chloride coordinated ions are located much further from the ruthenium atom (2.37 Å) than the two nitrogen atoms (2.11 Å and 2.13 Å). A possible explanation is related to the notable nucleophilic character of the chelating ligand that induces a notable increasing of the distance Ru-Cl. Furthermore, the bulky chelating ligand might be the origin of the slight reduction observed in the Cl-Ru-Cl angle value (91.4°), in comparison with the same angle in cisplatin (91.9°). These two effects have also been found in similar complexes with other aminopolycarboxylic ligands, *i.e.*, $Ru(edta)Cl_2$ [22] and $Ru(pdta)Cl_2$ [23].

Clearly, the distance Ru-O(11) is shorter (2.03 Å) than the distance Ru-O(21) (2.07 Å). As a consequence, the octahedral configuration around ruthenium atom is slightly distorted (Fig. 1) with angles

**Table 2**

Selected bond lengths [A] and angles [deg] for $[Ru(H_2cdta)Cl_2]\cdot 2H_2O$

| | |
|---|---|
| Ru(1)-O(11) | 2.028(3) |
| Ru(1)-O(21) | 2.070(3) |
| Ru(1)-N(1) | 2.108(2) |
| Ru(1)-N(2) | 2.128(2) |
| Ru(1)-Cl(2) | 2.3658(9) |
| Ru(1)-Cl(1) | 2.3723(8) |
| O(11)-Ru(1)-O(21) | 173.48(8) |
| O(11)-Ru(1)-N(1) | 80.72(9) |
| O(21)-Ru(1)-N(1) | 94.89(8) |
| O(11)-Ru(1)-N(2) | 95.57(9) |
| O(21)-Ru(1)-N(2) | 79.14(10) |
| N(1)-Ru(1)-N(2) | 84.54(8) |
| O(11)-Ru(1)-Cl(2) | 93.05(7) |
| O(21)-Ru(1)-Cl(2) | 91.97(7) |
| N(1)-Ru(1)-Cl(2) | 92.69(7) |
| N(2)-Ru(1)-Cl(2) | 170.39(8) |
| O(11)-Ru(1)-Cl(1) | 91.83(6) |
| O(21)-Ru(1)-Cl(1) | 92.21(6) |
| N(1)-Ru(1)-Cl(1) | 171.69(8) |
| N(2)-Ru(1)-Cl(1) | 92.57(6) |
| Cl(2)-Ru(1)-Cl(1) | 91.39(3) |

significantly reduced from ideal octahedral values, *i.e.*, N(1)-Ru-N(2), 84.5°; O(11)-Ru-N(1), 80.7° and O(21)-Ru-N(2), 79.1°.

It is important to remark that the nonbonding Cl...Cl distance (bite) in compound **1** (3.35 Å), is directly correlated with the distance between adjacent or proximal coordination sites in biological targets such as DNA. This bite distance is identical to that for cisplatin, and corresponds to the separation between two appropriate DNA-nucleobase donor atoms, this fact enabling cross-linking formation after interaction of complex **1** with DNA inside the cell /29/.

In the packing, the molecules of complex **1** are assembled in a 3D-network by intermolecular hydrogen bonding that involves two pairs of oxygen atoms, $O(13)\cdots O(24^1)$, 2.65 Å and $O(23)\cdots O(14^2)$, 2.67 Å (symmetry transformations; 1: $x-y+1, x, z-1/6$ and 2: $y, -x+y+1, z+1/6$), as found in similar cdta complexes /42a/, *i.e.*, the hydroxyl groups of the non-coordinated carboxylic moiety act as hydrogen-atom donors while the carbonyl groups are the hydrogen-atom acceptors in these associations (Fig. 2). In addition, the two water molecules located between neighbouring molecular units are also H-bonded to oxygen carboxylato groups {$O(1)\cdots O(21^3)$, 2.89 Å and $O(2)\cdots O(11^3)$, 2.87 Å (symmetry transformations; 3: $y, -x+y, z+1/6$)}. This H-bonded pattern can be described as a network of supramolecular helices. In the *c* direction the packing generates a sixfold screw axis and parallel channels (Fig. 3), with cavities of radius 2.15 Å. These channels are hydrophilic because the oxygen atoms of carboxylic moieties and those of water molecules point towards the cavities.

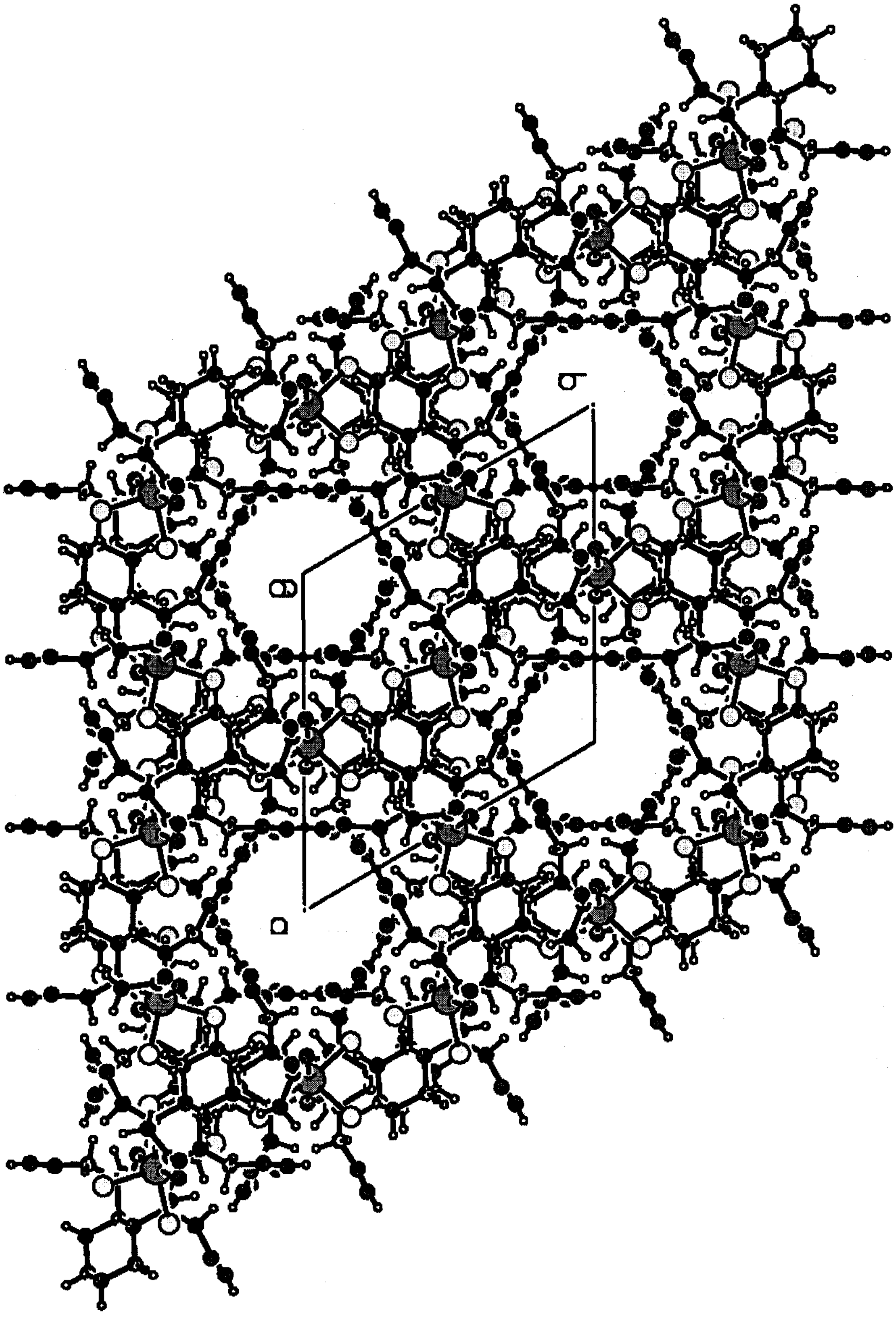

Fig. 2: Packing drawing view of 1 showing the assembling of molecules in a 3D-network by intermolecular hydrogen bonding involving two pairs of oxygen atoms.

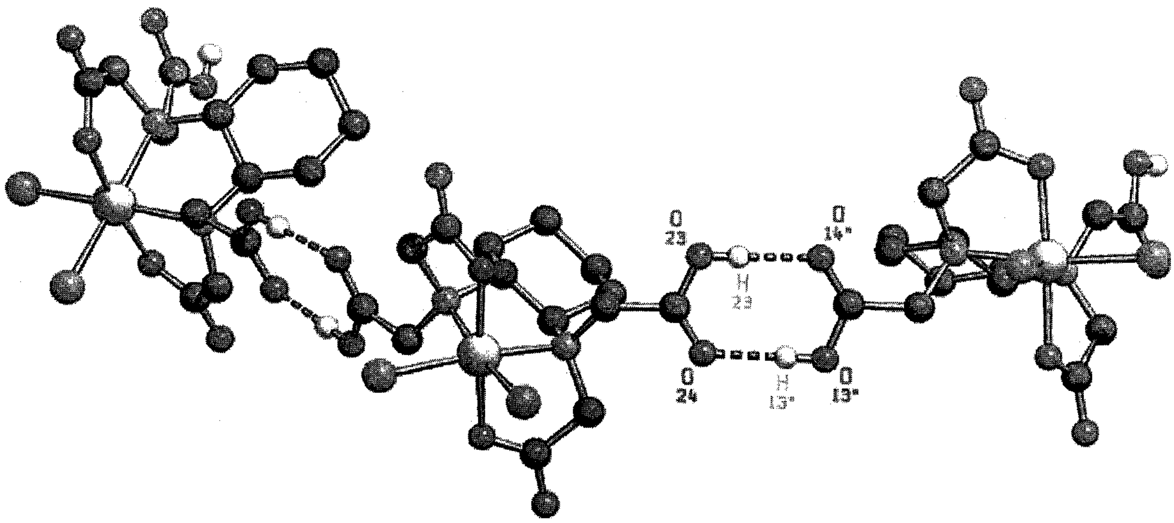

**Fig. 3:** CPK model view of the molecular packing along the *c*-axis showing the channels (water molecules located within the channels have been omitted for clarity).

### Potentiometric and conductimetric study

The potentiometric titration of an aqueous solution of the complex **1** (Fig. 4a) shows a sharp increase of pH for the consumption of 2 g-equiv. of alkali with an inflection point registered at pH 6.9. Furthermore, a change in the electrical conductivity of the solution is also observed at $\Lambda$ equal to 168 S cm$^2$ (Figure 4b),

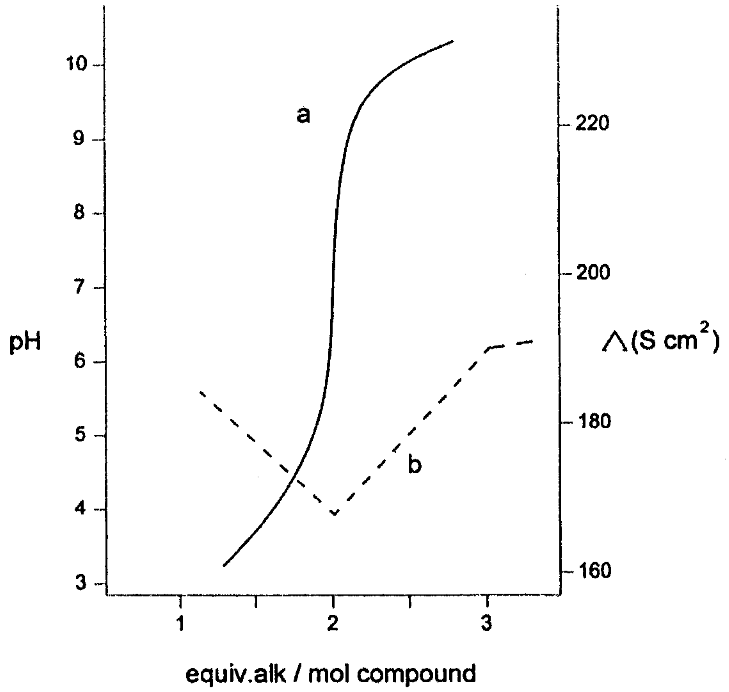

**Fig. 4:** Potentiometric (curve a) and conductimetric (line b) study of complex **1**.

these facts indicating the simultaneous neutralization of two free-COOH groups of the same strength. These results supported the tetradentate character for the cdta, which contains two coordinated carboxylate moieties and two free carboxylic groups. Molecular weight deduced from the titration corresponds closely with the expected for the dehydrated complex (MW: 516).

### Ultraviolet and visible spectroscopy

In the electronic spectrum (not shown) three different absorptions bands at 28410 $cm^{-1}$ (352 nm; $\varepsilon$ = 11310 $dm^3$ $mol^{-1}$ $cm^{-1}$), 31056 $cm^{-1}$ (322 nm; $\varepsilon$ = 15600 $dm^3$ $mol^{-1}$ $cm^{-1}$) and 35336 $cm^{-1}$ (283 nm; $\varepsilon$ = 4070 $dm^3$ $mol^{-1}$ $cm^{-1}$) are observed. The first two main bands could be attributed to the electronic transitions $^2E_g \leftarrow {}^3T_{1g}$ and $^3T_{2g} \leftarrow {}^3T_{1g}$, respectively, while the smaller band (shoulder) registered at 283 nm could be due to the transition $^3A_{1g} \leftarrow {}^3T_{1g}$. This behaviour suggests a low spin $d^4$ system, normally presenting two close bands of similar intensities originated by transitions from unpaired basal states.

### Infrared spectroscopy

The FT-infrared spectrum of compound **1** (not shown) presents a broad, split signal with main peaks at 2940 $cm^{-1}$ and 2902 $cm^{-1}$ corresponding to the symmetric stretching vibrations ($\nu_s$) of the methylene groups (C-H bonds) contained in the heterocyclic ligand and of the same groups next to coordinated and free carboxylic groups. The O-H stretching vibrations of the free COOH groups are responsible for the broad absorptions registered between 3350 $cm^{-1}$ and 2600 $cm^{-1}$. The complex shows strong characteristic bands registered at 1735 $cm^{-1}$ ($\nu_{as}$, C=O bond of free carboxylic groups) and 1610 $cm^{-1}$ ($\nu_{as}$, $COO^-$ coordinated carboxylate groups) whereas the $\nu_s$ of coordinated carboxylate groups appeared at 1400 $cm^{-1}$. The difference of 210 $cm^{-1}$ between $\nu_{as}$ and $\nu_s$ of coordinated carboxylate groups indicates the predominantly ionic character of complex **1**.

Water normally gives broad absorption at about 3400 $cm^{-1}$. Complex **1** shows indeed a broad and intense band around 3350 $cm^{-1}$ that might be attributed to the hydration water molecules most likely bonded to anionic coordinated moieties through hydrogen bonds /55/. Table 3 presents the main bands observed in the infrared spectrum of complex **1**.

### Cyclic voltammetry

The cyclic voltammogram (CV) of just prepared 4.0 mM aqueous solutions (pH 2.0) of complex **1** ($NaClO_4$, 0.15 M) is presented in Fig. 5. At electric potentials between +0.5 V and -0.5 V, the CV show a cathodic wave (A) immediately followed by the anodic wave (A'), both clearly constituting a coupled pair, AA'. If the potential varies between +1.5 V and -1.5 V, an intense cathodic peak is observed at about - 0.8 V, while an anodic prominent peak appears at about 1.0 V. These features could be attributed to redox processes affecting the ligands /58/.

Table 4 presents the cyclovoltammetric parameters corresponding to the coupled pair AA', characteristic

**Table 3**

IR bands ($cm^{-1}$) for complex $[Ru(H_2L)Cl_2]\cdot 2H_2O$

| COOH | COO⁻ | $CH_2$ (C-H) | $H_2O$ (hyd) | Ru-N | Ru-Cl |
|---|---|---|---|---|---|
| 1735 $\nu_{as}$ | 1610$\nu_{as}$ | 2940 | 3550$\nu_s$ | 540 | 240 |
| 3350-2600 | 1400$\nu_s$ | 2902 | 1630$\nu_b$ | 1100 | 220 |

$\nu_s$ and $\nu_{as}$: symmetric and asymmetric stretching vibrations; $\nu_b$: bending vibration. See references /56,57/ for vibrations Ru-N and Ru-Cl.

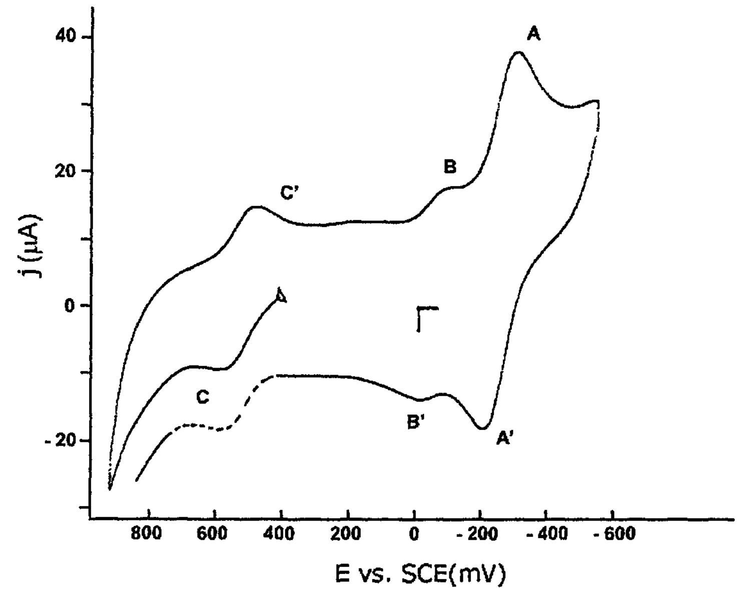

**Fig. 5:** Cyclic voltammogram of complex 1 (4 mM). Potentials measured against GCE ($NaClO_4$ 0.15 M); pH, 2.02.

of a reversible one-electron process (diffusive control), in agreement with the values of the cathodic current function, in the order of 300 A cm $mol^{-1}$ $s^{1/2}$ $V^{-1/2}$. The pair AA' of just prepared solutions of complex 1 is always preceded by another less intense pair (BB', $E_f$ = - 50 mV ) coupled with a reversible third pair CC' at positive potential values (*i.e.*, $E_f$ = + 535 mV; see Fig.5). Different coexisting Ru(III) species seem to be responsible for the processes AA' and BB', as suggested by the invariance of the used scanning speed interval. However, the process BB' is the origin of the coupled process CC' (initial scanning in anodic direction), the last probably due to the oxidation Ru(III)-Ru(IV). In fact, two reversible/quasi-reversible couples at - 0.25 V and 0.54 V are observed, characteristic of the one-electron reduction processes Ru(III)/Ru(II) and Ru(IV)/Ru(III), respectively, in agreement with previous structural results shown in this research.

**Table 4**

Cyclovoltammetric parameters (pair AA')

| v (V/s) | $E_{pc}$ | $E_{pa}$ | $E_f$ | $E_{pc}$-$E_{pa}$ | $E_p$-$E_{p/2}$ | $I_{pc}$ |
|---|---|---|---|---|---|---|
| 0.05 | - 280 | - 220 | - 250 | 60 | 65 | 305 |
| 0.08 | - 285 | - 215 | - 250 | 70 | 65 | 300 |
| 0.10 | - 290 | - 215 | - 252 | 75 | 70 | 210 |
| 0.12 | - 290 | - 210 | - 250 | 80 | 75 | 300 |
| 0.15 | - 290 | - 210 | - 250 | 80 | 80 | 305 |
| 0.17 | - 295 | - 210 | -252 | 85 | 80 | 300 |
| 0.20 | - 300 | - 200 | - 250 | 100 | 90 | 310 |

Complex **1** solution (4.0 mM) in $NaClO_4$ 0.15 M. Potential values in mV. Current function, $I_{pc}$ ( = $i_{pc}/Ac\sqrt{v}$) in A cm $mol^{-1}$ $s^{½}$ $V^{-½}$.

Table 5 shows the peak and formal electric potentials obtained at different acid pH values. Addition of NaOH(aq) or HCl(aq) does not cause significant alterations of the CV patterns, but lesser negative potentials are measured if pH decreases. This result is consistent with the existence of possible ligand protonation equililibria.

**Table 5**

Peak and formal potential values (mV) for the AA' pair at different pH values

| pH | $E_{pc}$ | $E_{pa}$ | $E_f$ |
|---|---|---|---|
| 2.02 | - 290 | - 210 | - 250 |
| 1.94 | - 295 | - 165 | - 230 |
| 1.38 | - 270 | - 150 | - 210 |

### Cytotoxic and antiproliferative behaviour

The inhibiting effects of $[Ru(H_2L)Cl_2]\cdot 2H_2O$ against human uterine neck carcinoma cell line (HeLa) are shown in Fig. 6, which presents the evolution with time of the total number of culture cells in the absence (**control** curve) or presence of increased amounts of the tested compound (curves **a** and **b**). After three days of treatment, a ruthenium complex dose of 25.4 μM (curve **a**) slowed cell proliferation in about 27% if compared with control and this cell inhibition keeps constant during the remaining days of treatment. If the dose is increased to 40.8 μM, the antiproliferative activity of complex **1** is significantly enhanced not only on the third day of treatment (47% of inhibition) but also during the three following days (curve **b**; *i.e.*, 55% at fourth day), as manifested by the smooth ascendant slope of the curve at this drug concentration. These results show that $[Ru(H_2L)Cl_2]\cdot 2H_2O$ is remarkably cytotoxic against HeLa human cells at dose of 40.8 μM.

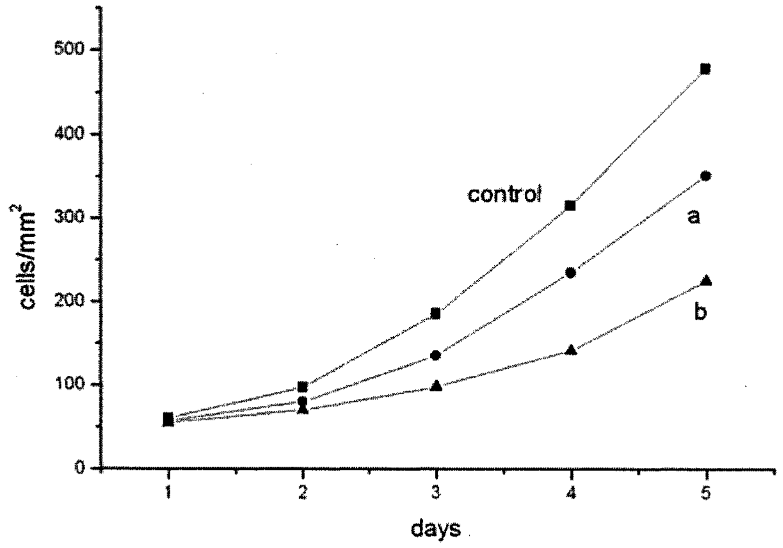

**Fig. 6:** Antiproliferative effects exerted by the $[Ru(H_2L)Cl_2]\cdot 2H_2O$ complex on the grow kinetics of the human uterine neck carcinoma HeLa. Cells were exposed to several doses of the ruthenium complex for a total of five days, as indicated: **control** curve (untreated HeLa cells); curve **a** (25.4 μM); curve **b** (40.8 μM).

To determine if this cytotoxic behaviour holds true for other human tumours, the inhibition assay was repeated against the human malign melanoma ADLD (Fig. 7) whose proliferation rate (**control** curve) is higher than that observed for HeLa cells. A substantial increase of the antiproliferative properties was detected against this melanoma at the same doses assayed against HeLa carcinoma. At a dose of 25.4 μM, (curve **a**) the inhibition of ADLD cells is not only more important that in the previous assays but increases with increasing time (inhibition of about 33% after 72 h; 42.4% on the fourth day, and 46.4% on the last day). If the dose is increased (40.8 μM) the same general pattern is observed (increasing cytotoxicity at increasing time) and the complex **1** shows important antiproliferative response next to 70% during the last two days of treatment (curve **b**).

Fig. 8 shows the antiproliferative behaviour of complex **1** against the colon adenocarcinoma cell line HT-29. As can be seen, the cell growth observed in this culture (**control** curve) is slightly slower than HeLa cell culture while the cytotoxic study indicates that ruthenium complex exhibits discrete cell inhibition activity at both doses assayed. The analysis of the antiproliferative curve at the low dose (25.4 μM, curve **a**) shows that ruthenium complex behaves in a similar way to that analyzed for HeLa cells (the inhibition remains constant after three days of treatment), but the antiproliferative activity decreased to about 20%, if compared to that observed at this dose for HeLa cells (27%). When the dose is increased (40.8 μM, curve **b**), the observed cell inhibition after 72 h of treatment is enhanced to 25%. Interestingly, this inhibition also remains constant during the treatment.

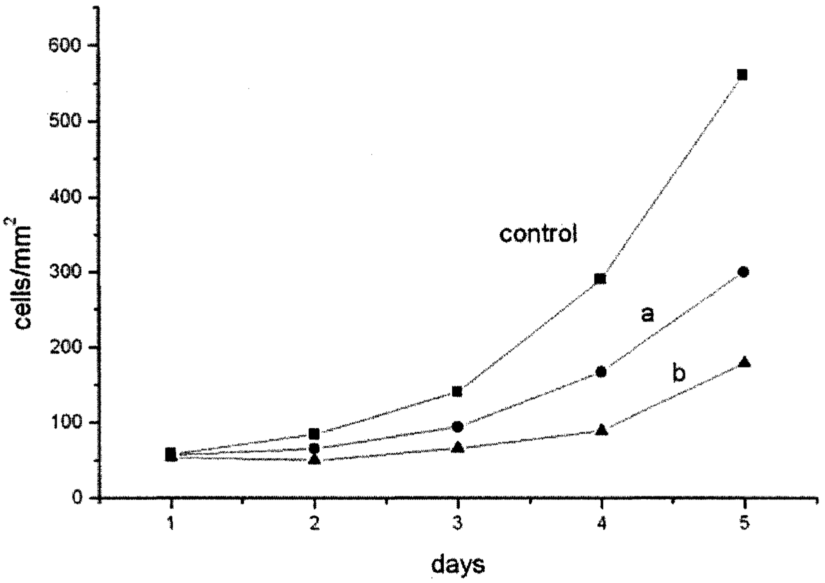

**Fig. 7:** Antiproliferative effects exerted by complex **1** on the grow kinetics of the human malignant melanoma (ADLD). Cells were treated with the complex for a total of five days, as indicated: **control** curve (untreated ADLD cells); curves **a** and **b** correspond to the treatment of cells at same doses as Fig. 6.

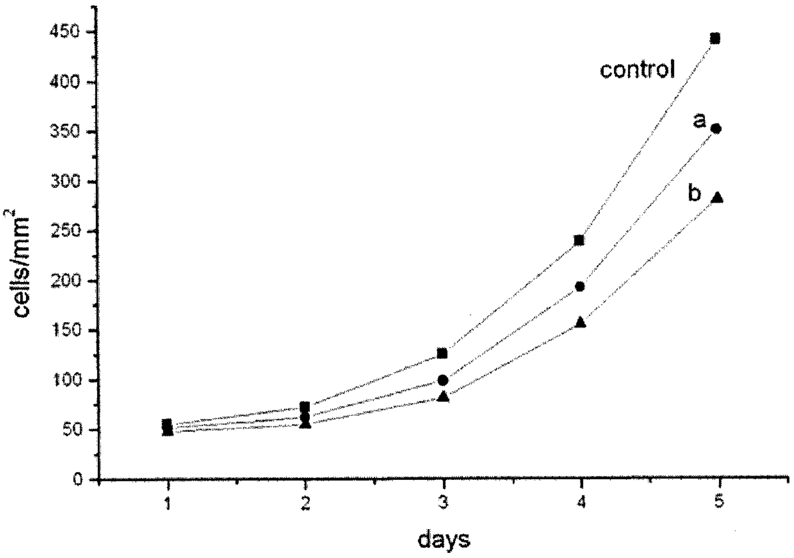

**Fig. 8:** Antiproliferative effects exerted by complex **1** on the grow kinetics of the human colon adenocarcinoma cancer HT-29. Cells were exposed to several doses of the ruthenium complex for a total of five days, as indicated: **control** curve (untreated HT-29 cells); curves **a** and **b** correspond to the treatment of cells at same doses as Fig. 6.

The *in vivo* antitumour activity of complex **1** against EAT and P388 tumours was evaluated at several treatment doses in the range 20–240 mg/kg body weight. The therapeutic activity of the complex was obtained from the T/C percentage which is described as T/C% = (100) x mean life span of treated mice/ mean life span of untreated mice; tumour free survivors were excluded. The minimum value of T/C for moderate activity is 120 (EAT and P388 tumours); if T/C > 125 the complex is considered a candidate for further antitumour assays. T/C values required for activity in MX-1 xenograft tumour must be lower than 20 (T/C <20).

For EAT, a T/C (%) of 350 was obtained (25 mg/kg) and the complete remission of the tumour observed at a dose of 50 mg/kg. The toxic dose ($LD_{50}$) was equal to 100 mg/kg. In the case of the lymphocytic leukemia P388, a T/C(%) value of 140 was obtained (60-120 mg/kg) while in MX-1 xenograft carcinoma, this parameter was equal to 16 at a dose of 240 mg/kg.

These results evidence a notable and specific antiproliferative effect of complex $[Ru(H_2L)Cl_2]\cdot 2H_2O$ against the human tumour cell lines ADLD and HeLa, with selective and important cytotoxic properties in both cases. The *in vivo* antitumour activity is also remarkable in the studied tumours. A further investigation of the antineoplastic activity of this new potential ruthenium drug against other types of tumour is advisable.

### ACKNOWLEDGEMENTS

We greatly appreciate financial support from MCYT, Spain (Grants PPQ2000-0035-P4 and BQU2001-2455) and EC (COST Chemistry Projects D20/005 and D20/009). The authors are grateful to Prof. A. Cervilla and V. Folgado, from Valencia University (Spain), for the help provided in the cyclovoltammetric study.

### REFERENCES

1. N. Farrell, in: *Platinum-based Drugs in Cancer Chemotherapy*, L.R. Kelland and N. Farrell (Eds.), Humana Press, Totowa, N.J., 2000; p. 321.
2. E.R. Jamieson and S.J. Lippard, *Chem.. Rev.*, **99**, 2467 (1999).
3. M.J. Bloemink and J. Reedijk, in: *Metal Ions in Biological Systems*, A. Sigel and H. Sigel (Eds.), vol. **32**, Dekker, New York, 1995; Chapter 19.
4. J. Reedijk, *PNAS*, **100**, 3611 (2003).
5. J. Reedijk, *Curr. Opin. Chem. Biol.*, **3**, 236 (1999).
6. Z.J. Guo and P.J. Sadler *Angew. Chem. Int. Edit.*, **38**, 1513 (1999).
7. E. Wong and C.M. Giandomenico, *Chem. Rev.*, **99**, 2451 (1999).
8. G. Giaccone, *Drugs*, 59, Suppl. 4-9 (2000).
9. S.L. Bruhn, J.H. Toney and S.J. Lippard, *Prog. Inorg. Chem.*, **38**, 477 (1990).
10. A. Eastman, in: *30 Years of Cisplatin: Chemistry and Biochemistry of a Leading Anticancer Drug*, B. Lippert (Ed.), Verlag Helvetica Chimica Acta, Zürich, 1999, p. 111.

11. J.A.R. Navarro, J.M. Salas, M.A. Romero, R. Vilaplana, F. González-Vílchez and R. Faurè, *J. Med. Chem.*, **41**, 332 (1998).

12. M. Galanski, M. Berger and B.K. Keppler, *Metal-Based Drugs*, **7**, 349 (2000).

13. A. Zenker, M. Galanski, T.L. Bereuter, B.K. Keppler and W. Lindner, *J. Biol. Inorg. Chem.*, **5**, 498 (2000).

14. B. Desoize and C. Madoulet, *Crit. Rev. Oncol. Hematol.*, **42**, 317 (2002).

15. N. Farrell, Y. Qu, L. Feng and B. Van Houten, *Biochemistry* **29**, 9522 (1990).

16. K. Akdi, R. Vilaplana, S. Kamah, J.A.R. Navarro, J.M. Salas and F. González- Vílchez, *J. Inorg. Biochem.*, **90**, 51 (2002) and ref. therein.

17. B.K. Keppler, *New J. Chem.*, **14**, 389 (1990).

18. M.J. Clarke, F. Zhu and D.R. Frasca, *Chem. Rev.* **99**, 2511 (1999).

19. G. Sava, A. Bergamo, S. Zorzet, B. Gava, C. Casarsa, M. Cocchietto, A. Furlani, V. Scarcia, B. Serli, E. Iengo, E. Alessio and G. Mestroni, *Eur. J. Cancer*, **38**, 427 (2002).

20. M.J. Clarke, *Coord. Chem. Rev.*, **236**, 209 (2003).

21. F. González-Vílchez and R. Vilaplana, *Rev. Biol. Cel.*, **53**, 194 (1986).

22. R. Vilaplana, M.G. Basallote, C. Ruiz, E. Gutierrez-Puebla and F. González- Vílchez, *J. Chem. Soc., Chem. Común.*, 100 (1991).

23. R. Vilaplana, M.A. Romero, M. Quirós, J.M. Salas and F. González-Vílchez, *Metal-Based Drugs*, **2**, 211 (1995).

24. S.R. Grguric-Sipka, R.A. Vilaplana, J.M. Pérez, M.A. Fuertes, C. Alonso, Y. Alvarez, T.J. Sabo and F. González-Vílchez, J. Inorg. Biochem., **97**, 215 (2003).

25. R. Vilaplana, F. González-Vílchez, F. Delmani, J. Torreblanca, J. Moreno and G. García-Herdugo, *J. Biol. Inorg. Chem.*, (submitted).

26. F. González-Vílchez, R. Vilaplana, G. Blasco and L. Messori, *J. Inorg. Biochem.*, **71**, 45 (1998).

27. E. Gallori, C. Vettori, E. Alessio, F. González-Vílchez, R. Vilaplana, P. Orioli, A. Casini and L. Messori, *Arch. Biochem. Biophys.*, **376**, 156 (2000).

28. L. Messori, F. González-Vílchez, R. Vilaplana, E. Piccioli, E. Alessio and B.K. Keppler, *Metal-Based Drugs*, **7**, 335 (2000).

29. M. Carballo, R. Vilaplana, G. Márquez, M. Conde, F.J. Bedoya, F. González- Vílchez and F. Sobrino, *Biochem. J.*, 328, 559 (1997).

30. D.J. Sánchez, M. Gómez, J.L. Domingo and J.M. Llobet, *J. Appl. Toxicol.*, **15**, 285 (1995).

31. M.D. Sousa-Neto, J.G. Passarinho, J.R. Carvalho, A.M. Cruz, J.D. Pécora and P.C. Saquy, *Braz. Dent. J.*, **13**, 123 (2002).

32. C. Rihouey, C. Morvan I. Borissova, A. Jauneau, M. Demarty and M. Jarvis, *Carbohydrate Polymers*, **28**, 159 (1995).

33. A.I. Valle, M.J. González and M.L. Marina, *J. Chromat.*, **607**, 207 (1992); E.A. Gautier, R.T. Gettar, R.E. Servant and D.A. Batistoni, *J. Chromat.*, **706**, 115 (1995); M.C. Breadmore, M. Mack and P.R. Haddad, *Anal. Chem.*, **71**, 1826 (1999).

34. G. Schwarzenbach and H. Ackermann, *Helv. Chim. Acta*, **32**, 1682 (1949); J.H. Holloway and C.N. Reilley, *Anal. Chem.*, **32**, 249 (1960); D. Wright, J.H. Holloway and C.N. Reilley, *Anal. Chem.*, **37**, 384

(1965); J.D. Carr and D.G. Swartzfager, *Anal. Chem.*, **43**, 1520 (1971); E. Carmona and F. González, *Anal. Quím.*, **72**, 768, 773 (1976).

35. D. Atzey, D. Defilippo, A. Rossi and R. Caminiti, *Spectrochim. Acta (A)*, **49**, 1779 (1993).
36. J.D. Martín, J.M. Tercero, A. Matilla, J. Niclós, A. Busnot and S. Ferrer, *Polyhedron*, **15**, 439 (1996).
37. S. Seibig and R. van Eldik, *Inorg. Chim. Acta*, **279**, 37 (1998); *Eur. J. Inorg. Chem.*, 447 (1999).
38. T. Schneppensieper, S. Seibig, A. Zahl, P. Tregloan and R. van Eldik, *Inorg. Chem.*, **40**, 3670 (2001).
39. K.Y. Choi, K.S. Kim and C.P. Hong, *Bull. Kor. Chem. Soc.*, **15**, 782 (1994).
40. E. Szilagyi and E. Bruecher, *J. Chem. Soc. Dalton Trans.*, **13**, 2229 (2000).
41. S.P. Petrosyants and A.B. Ilyukhin, *Russ. J. Inorg. Chem.*, **47**, 712 (2002).
42. a) E.N. Duesler, R.E. Tapscott, M.G. Basallote and F. González-Vílchez, *Acta Cryst.*, **41C**, 678 (1985); b) M.G. Basallote, R. Vilaplana and F. González- Vílchez, *Polyhedron*, **13**, 1853 (1994). c) J.M. López-Alcalá, M.C. Puerta and F. González-Vílchez, *Rev. Chim. Minèrale*, **21**, 257 (1984); d) F. González-Vílchez, M.G. Basallote, J. Benítez and R. Vilaplana, *Rev. Esp. Oncol.*, **29**, 609 (1982).
43. M.M. Taqui Khan, A. Kumar and Z. Shirin, *J. Chem. Res. Mp.*, **4**, 1001 (1986).
44. M.M. Taqui Khan, R.C. Bhardwaj, C. Bhardwaj and N.N. Rao, *J. Photochem. Photobiol. A-Chemistry*, **68**, 137 (1992).
45. T.V. Filippova, T.N. Polyona, A.L. Il'inskii, M.A. Porai-Koshits and N.A. Ezerskaya, *Zh. Neorg. Khim.*, **26**, 1418 (1981); A preliminary communication on the structure of this complex was previously published: R. Vilaplana, F. González- Vílchez, E. Gutierrez-Puebla and C. Ruiz-Valero, *Inorg. Chim. Acta*, **224**, 15 (1994).
46. B.V. Nonius, CAD4 Express Software, Ver. 5.1/1.2. Enraf Nonius, Delft, The Netherlands (1994).
47. M. Kretschmar, GENHKL Program for the reduction of CAD4 Diffractometer data, University of Tuebingen, Germany (1997).
48. A.C.T. North, D.C. Phillips and F.S. Mathews, *Acta Cryst.* **24A**, 351 (1968).
49. G.M. Sheldrick, *Acta Cryst.*, **46A**, 467 (1990).
50. G.M. Sheldrick, SHELXL-97, Program for the Refinement of Crystal Structures, University of Goettingen, Germany (1997).
51. A.L. Spek, PLATON, A Multipurpose Crystallographic Tool, Utrecht University, Utrecht, The Netherlands (2002).
52. H.D. Flack, *Acta Cryst.*, **39A**, 876 (1983).
53. International Tables for Crystallography, Vol. C, Kluwer Academic Publishers: Dordrecht, The Netherlands (1995).
54. E. Keller, SCHAKAL-97, A computer program for the graphic representation of molecular and crystallographic models. University of Freiburg i. Br., Germany (1997).
55. J. Fujita, K. Nakamoto and M. Kobayashi, *J. Amer. Chem. Soc.*, **78**, 3963 (1958).
56. P.C. Kong and F. Rochon, *Can. J. Chem.*, **57**, 526 (1979)
57. B. de Klerk-Engels, H.-W. Frühauf and K. Vrieze, *Inorg. Chem.*, **32**, 5528 (1994).
58. A.A. Diamantis and J.V. Dubrawsky, *Inorg. Chem.*, **22**, 1934 (1983).

